# Recurrent Pelvic Inflammatory Disease After Serial Intrauterine Inseminations

**DOI:** 10.1155/crog/1426202

**Published:** 2025-06-22

**Authors:** Lubos Karasek, Pavla Svobodova, Imrich Kiss, Jan Smetana

**Affiliations:** ^1^Department of Gynecology 3rd Faculty of Medicine of Charles University and Military University Hospital Prague, Military University Hospital Prague, Prague, Czech Republic; ^2^Department of Epidemiology, Military Faculty of Medicine, University of Defence, Hradec Kralove, Czech Republic

**Keywords:** acute pelvic pain, intrauterine insemination, pelvic inflammatory disease, sexually transmitted infection, sterility

## Abstract

**Introduction:** Intrauterine insemination is a basic method of assisted reproduction. It enables direct deposition of sperm inside the uterine cavity. The complications are rare. Multiple pregnancy or ovarian hyperstimulation can occur when concomitant ovulation induction is performed. The risk of pelvic inflammatory disease due to the artificial cervical barrier breach is very low, but the possible consequences are serious.

**Case presentation:** We present a case of recurrent pelvic inflammatory disease following consecutive intrauterine inseminations. Pelvic inflammatory disease pathophysiology and treatment approaches are discussed.

**Conclusion:** Inflammatory complications of the IUI are rare, but the possible impact on fertility can be devastating. Prevention of the PID together with prompt diagnosis and individualized therapy should be assured to preserve fertility.

## 1. Introduction

Intrauterine insemination (IUI) is one of the essential methods in infertility treatment. It enables deposition of concentrated sperm directly into the uterine cavity [1]. IUI is considered a first-line of assisted reproductive technology (ART) therapy for patients with functionally normal tubes and infertility due to cervical factor or moderate male factor. The IUI is a technically simple procedure with an extremely low rate of complications. However, access to the uterine cavity through the physiologic cervical mucosal barrier can facilitate migration of vaginal microorganisms into the upper genital tract. Pelvic inflammatory disease (PID) is a severe complication that can lead to deterioration of fertility, permanent adnexal damage, and even life-threatening situation in case of tubo-ovarian abscess [2]. The reported post-IUI PID rate from large IUI registers is low, ranging from 0 to 0.17/1000 IUI cycles [3]. The risk of PID is increased in patients with multiple sexual partners, history of PID or surgical procedures on the upper genital tract.

In this report, a case of a female with recurrent PID following multiple IUI is described, and possible pathophysiological implications are discussed. All described procedures were performed in compliance with relevant laws and institutional guidelines and have been approved by the appropriate institutional ethics committee.

## 2. Patient Information

**Figure 1:**
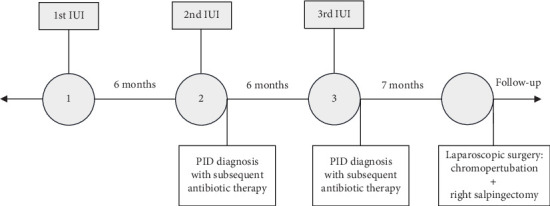
Timeline of the case.

A 32-year-old female presented to an emergency unit of the tertiary healthcare facility with acute severe lower abdominal pain and fewer exceeding 38°C approximately 24 h after her second IUI attempt (Figure 1).

The patient was considered a healthy individual and used no chronic medication prior the incident. No serious or hereditary disease was recorded in her family history.

The patient had undergone a conisation for low-grade cervical dysplasia 7 years ago and had been treated for primary sterility for the last 3 years. She underwent the first unsuccessful IUI in a primary health care facility 6 months before the reported incident. No sign of inflammatory complications was reported. The second IUI was performed in a primary healthcare facility again. The patient was tested negative for STIs before the IUI and underwent Clomiphene citrate ovulation induction. The gynecological examination and vaginal sonography performed before and during the induction were completely physiological. Detailed examination of fallopian tube patency, like hysterosalpingography, contrast ultrasonography, or laparoscopic chromopertubation, was not performed prior the IUI attempts. No bacteriological cultures from partner's urine were obtained before the IUI. The semen was prepared following the internal protocol using a gradient-wash technique, and IUI was performed without complications on Day 14 of the menstrual cycle.

## 3. Clinical Findings

Initial examination at the emergency unit revealed a body temperature of 37.9°C. Other vital signs were normal. Severe palpable soreness over the whole hypogastrium with defense musculaire was present at the physical examination (PE). Gynecological speculum exam showed a thick vaginal secretion. Overall pelvic tenderness with painful cervical motion was noted on bimanual vaginal examination.

## 4. Diagnostic Assessment

The most significant findings on PE were signs of peritoneal irritation and fever. Blood test analysis showed inflammatory marker elevation. White blood cell count (WBC) 20 × 10^9^/L, C-reactive protein (CRP) 186 mg/L, and procalcitonin (PCT) 0.393 *μ*g/L. Bacterial smear for cultivation and STI polymerase chain reaction (PCR) diagnostics was collected from the cervix during the gynecological examination.

Imaging survey was provided by transvaginal sonography, revealing bilaterally enlarged ovaries corresponding with ovulation induction and no other pelvic pathology. Abdominal sonography showed a normal appendix without signs of irritation.

Considering all clinical, laboratory, and imaging findings, diagnosis of PID following the IUI was made. With normal sonographic findings, diagnoses like appendicitis or bowel inflammatory disease were less probable.

## 5. Therapeutic Intervention

The patient was hospitalized, and empirical intravenous (IV) antibiotic therapy was started with ampicillin + sulbactam 3 g IV every 6 h, following a hospital antibiotics protocol. A peak level of CRP 256 mg/L was recorded on the second day of the therapy. Rapid clinical improvement and decrease in inflammatory markers were observed from the third day of the therapy. The IV therapy was held for 8 days ensuring an improvement in the clinical status of the patient. There was no need of the antibiotic regiment change because great clinical improvement was observed, and no conclusive pathogen was revealed in cervical smears.

**Figure 2:**
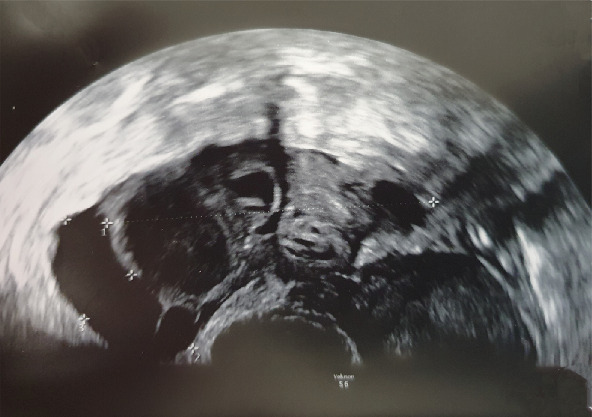
Transvaginal sonography finding in the right adnexal region after the first PID episode.

The sonography at the time of discharge from the hospital revealed a mixed echogenic mass in the right adnexal area, measuring 74 × 40 15 mm (Figure 2). The IUI was unsuccessful with null human chorionic gonadotropin (hCG) levels.

Cervical cultures and STI PCR were both negative. Nevertheless, the oral ampicillin + sulbactam therapy to finish 14 days regimen and subsequent oral doxycycline 10 days therapy was recommended taking into consideration the rapid PID course.

## 6. Follow-Up

An early follow-up appointment was arranged to reevaluate the clinical status. During the examination a month after the PID flare-up, no complaints were reported by the patient and no pathological findings on transvaginal sonography, except a small amount of pelvic fluid, were observed. Thus, a conservative approach was recommended.

The patient underwent a third IUI attempt 6 months after the PID incident. This time, the IUI was performed in a tertiary ART facility. Both partners were tested negative for STIs again. Fallopian tube patency test was not performed. An internal protocol for the semen processing using a magnetic-activated cell sorting method was followed.

**Figure 3:**
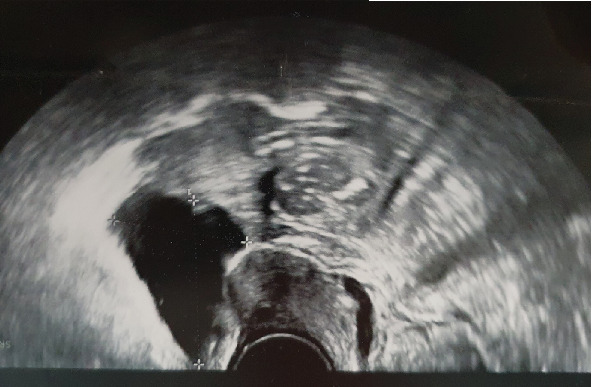
Transvaginal sonography findings in the left adnexal region after the second PID episode.

Approximately 24 h after the third IUI, the patient presented to an emergency unit with similar complaints and as serious clinical picture as 6 months earlier. Patient's fever reached 38.2°C, and PE showed severe tenderness in the lower abdominal quadrants with signs of peritoneal irritation. Gynecological examination revealed painful cervix movements on bimanual vaginal examination. Laboratory analysis showed extensively increased inflammatory markers with WBC 16 × 10^9^/L, CRP 141 mg/L, and PCT 0.8 *μ*g/L. Transvaginal sonography revealed a normal finding of the uterus and right ovary. There was an anechoic mass measuring 52 × 28 × 25 mm in the left adnexal region (Figure 3).

Diagnosis of recurrent PID with left sactosalpinx was stated. The patient was admitted to the hospital and empirical antibiotic therapy with ampicillin + sulbactam 3 g IV every 6 h was started, again with an excellent clinical response. Quick relief from the complaints and a decrease in inflammatory markers were observed through the 7 days of the IV antibiotic therapy. Again, no pathological results were retrieved from the cervical smears taken before the therapy.

Examination before the discharge from the hospital confirmed the persistent sactosalpinx in the left adnexal region measuring 52 × 32 × 25 mm and negative hCG levels. Additional oral ampicillin + sulbacatam and doxycycline treatment were administered.

Considering patient's wishes and will to conceive, further steps toward a fertility restoration were discussed. Delayed diagnostic hysteroscopy, endometrial biopsy, diagnostic laparoscopy, and assessment of tubal patency were recommended in cooperation with the patient's ART fertility center.

An examination before the planned surgery was performed 7 months after the second PID incident. The patient had no difficulties and felt healthy. Sonography revealed normal pelvic anatomy except a small anechoic structure in the left adnexal region. The hysteroscopy was unremarkable, as was the endometrial biopsy. The laparoscopy showed fine adhesions in the small pelvis region with the normally sized uterus fixed to the bladder and both ovaries fixed to the pelvic wall at ovarian fossa. Except the adhesions, ovaries were appropriate in size and appearance. Both fallopian tubes were dilated in the distal third to a maximum of 7 mm. Neither evident tubal adhesions nor clubbed fimbria was spotted bilaterally. Rectouterine pouch contained a small volume of serous fluid and was free of adhesions. The intestines were normal in appearance, including the appendix. No visible signs of endometriosis were seen in the abdominal cavity. Pelvic adhesions were dissected, and tubal patency test with methylene blue was performed with negative patency revealed in the right fallopian tube. Right salpingectomy was performed, following a preoperative agreement and consent of the patient. The patent tube on the left side remained in the pelvis. The postoperative period was calm without complications. Histology revealed fibrous changes in the right fallopian tube.

The patient was without any complications or difficulties and the gynecological examination was completely normal at the follow-up 6 months postoperatively. Possible in vitro fertilization (IVF) options were discussed through the ART fertility center.

## 7. Discussion

As far as the authors are aware, there are no published cases of repeated PID complications following an IUI. The separate incidents are rare as shown by Matorras et al. in his review [3]. However, there are case reports available that discuss specific inflammatory post-IUI complications [4, 5].

The recommended treatment of PID should reflect the high infertility risk of PID and consider the severity of the infection and clinical presentation. Two treatment approaches are generally used. In mild-to-moderate cases, the oral or intramuscular antimicrobial therapy is preferred. Intravenous regimens are preferable in the inpatient treatment of advanced PID cases [6–8] (Table 1). The efficiency of the outpatient treatment strategy in mild-to-moderate cases was shown in the PEACH trial [9].

No officially recommended antibiotic regimen was used in the presented case which could be perceived as a limitation of the chosen therapeutic approach. This is due to the fact that the used hospital's antibiotics protocol follows the recommendations of the institution's antibiotic center. It should also be emphasized that the availability of antibiotics differs between countries and even between hospitals, for example, Doxycyclin IV is not available in the Czech Republic. The patient recovered well on the administered parenteral therapy, and additional oral therapy was provided.

In the presented case, a pathologically impassable fallopian tube was removed with patient's consent, following a discussion with a fertility center specialist. Surgical procedure is usually inevitable in severe PID complications like pyosalpinx or tubo-ovarian abscess. Lavage and drainage of the pelvis, adhesiolysis, and eventually resection of damaged tissues, including salpingectomy or oophorectomy, could be the necessary steps to resolve the complications [10].

IUI is a very useful and technically simple fertility-preserving procedure. European Society of Human Reproduction and Embryology Consortium reports there were 139 178 IUI cycles performed in 2020 [11]. The reported post-IUI PID rate from large IUI registers is low, ranging from 0 to 0.17/1000 IUI cycles [3]. This is roughly 40% higher than frequency in the general population [12]. The frequency of the post-IUI PID seems to be higher when the husband sperm is used (0.21/1000) compared to donor sperm (0.03/1000) (*p* < 0.05; OR 6.95) [3]. The deposition of the sperms inside the uterine cavity through a catheter bypasses the natural cervical mucosal barrier, thus enabling migration and dissemination of vaginal microflora inside the uterus. The PID is mostly caused by an ascension of mixed aerobic and anaerobic microflora. STIs like *Chlamydia trachomatis*, *Neisseria gonorrhoeae*, and *Mycoplasma genitalium* are also frequently involved in the PID [13, 14]. A standard protocol in all ART fertility centers includes testing both partners for STIs before the IUI to prevent the PID [15]. The patient was tested prior to both described IUI trials. The partner was tested before the second complicated IUI, but we were not able to retrieve the information about testing before the first PID episode.

Another reason for the increased PID risk after the IUI could be the timing of the procedure. IUI is performed just before ovulation, at the peak of the proliferative phase of the menstrual cycle, when the uterine contractions move toward the uterine fundus and fallopian tubes, as shown by Kunz et al. [16]. The disruption of the ovarian outer layer during the ovulation with an exposition of ovarian stroma can also facilitate the spread of pathogenic microorganisms. The increased risk of PID associated with disruption of anatomical barriers and tissues can be also caused by surgical interventions like hysteroscopy or laparoscopy before the IUI. Certain conditions, like endometriosis, increase the PID risk both by themselves and by interventions performed to solve them [4].

## 8. Conclusion

We presented a case of repeated PID following an IUI, where the combination of rapid antibiotic treatment and surgery showed to be successful. Possible causes of the PID were discussed, and thorough prevention of the PID should be emphasized considering the possible consequences. Diagnosis of PID must be prompt, and the patient's fertility plans and desires should be always heard when selecting the treatment method. Targeted antibiotic regimen and maximally conservative surgery when indicated are approaches preferred to preserve fertility.

## 9. Patient Perspective

The patient was undergoing IUI in an attempt to conceive. With the first episode of PID, the patient's main concern was to relieve pain and get fit to continue with conception efforts as soon as possible. After explaining the nature of PID during the hospitalization, the patient's interest shifted to the possibilities of preventing PID and minimizing chances of its recurrence. After the second episode of PID, together with gynecologist and ART specialist, the patient decided that the surgical component of the treatment would be appropriate for the definitive solution of the recurrent health issues. After the surgery, which went without complications, the patient felt good and relieved. Once recovered from the recurrent episodes of PID and surgery, the patient began to consider IVF methods as the next adequate step in conception efforts.

## Figures and Tables

**Table 1:** Pelvic inflammatory disease treatment guidelines.

	**2017 European guidelines**	**CDC guidelines 2021**	**Czech literature recommendation 2022**
Outpatient regimens	Ceftriaxone 500 mg IM single dose followed by doxycycline 100 mg PO twice daily with metronidazole 500 mg PO twice daily for 14 days or Ofloxacin 400 mg PO twice daily plus metronidazole 500 mg PO twice daily for 14 days (ofloxacin may be replaced by levofloxacin 500 mg once daily) or Moxifloxacin 400 mg PO once daily for 14 days	Ceftriaxone 500 mg IM single dose plus doxycycline 100 mg PO twice daily with metronidazole 500 mg PO twice daily for 14 days or Cefoxitin 2 g IM single dose plus doxycycline 100 mg PO twice daily with metronidazole 500 mg PO twice daily for 14 days or Other parenteral third-generation cephalosporin (e.g., ceftizoxime or cefotaxime) plus oral doxycycline 100 mg orally twice daily with oral metronidazole 500 mg twice daily for 14 days For persons weighing ≥ 150 kg, 1 g of ceftriaxone should be administered.	Doxycycline 100 mg orally twice daily for 14 days with oral metronidazole 500 mg twice daily for 14 days or Amoxicillin–clavulanic acid 625 mg orally three times daily followed by doxycycline 100 mg PO twice daily for 14 days or Azithromycin 1 g single dose PO, with possible repetition once a week (max. 4 weeks) or Ofloxacin 400 mg PO twice daily for 14 days

Inpatient regimens	Ceftriaxone 1 g IV/IM once daily plus doxycycline 100 mg IV twice daily (oral doxycycline may be used if tolerated) followed by doxycycline 100 mg PO twice daily with metronidazole 500 mg PO twice daily to complete 14 days or Clindamycin 900 mg IV three times daily plus gentamicin IV/IM (3–6 mg/kg as a single daily dose with renal monitoring) followed by either Clindamycin 450 mg PO four times daily to complete 14 days or Doxycycline 100 mg PO twice daily with metronidazole 500 mg PO twice daily to complete 14 days Alternative regimens: ofloxacin 400 mg IV twice daily plus metronidazole 500 mg IV three times daily for 14 days or ceftriaxone 500 mg IM single dose plus azithromycin 1 g PO single dose followed by a second dose of azithromycin 1 g PO after 1 week	Ceftriaxone 1 g IV/IM once daily plus doxycycline 100 mg PO/IV twice daily with metronidazole 500 mg PO/IV twice daily or cefotetan 2 g IV twice daily plus doxycycline 100 mg PO/IV twice daily or cefoxitin 2 g IV every 6 h plus doxycycline 100 mg PO/IV twice daily Alternative regimens: ampicillin–sulbactam 3 g IV every 6 h plus doxycycline 100 mg PO/IV twice daily or clindamycin 900 mg IV every 8 h plus gentamicin loading dose IV/IM (2 mg/kg body weight) followed by a maintenance dose (1.5 mg/kg body weight) every 8 h; single daily dosing (3–5 mg/kg body weight) can be substituted. After clinical improvement with parenteral therapy, transition to oral therapy with doxycycline 100 mg 2 times/day and metronidazole 500 mg 2 times/day is recommended to complete 14 days of antimicrobial therapy.	Ampicillin–sulbactam 1.5 g IV four times daily plus gentamycin 240 mg IV once daily or amoxicillin–clavulanic acid 1.2 g IV three times daily plus gentamycin 240 mg IV once daily or clindamycin 600–900 mg four times daily plus gentamycin 240 mg IV once daily After clinical improvement with parenteral therapy, transition to oral therapy with doxycycline 100 mg 2 times/day and metronidazole 500 mg 2 times/day is recommended to complete 14 days of antimicrobial therapy.

PO—orally IM—intramuscularly IV—intravenously			

## Data Availability

The data that support the findings of this study are available on request from the corresponding author. The data are not publicly available due to privacy or ethical restrictions.

## References

[1] Cohen M. R. (1962). Intrauterine Insemination. *International Journal of Fertility*.

[2] Westrom L. (1995). Effect of Pelvic Inflammatory Disease on Fertility. *Venereology*.

[3] Matorras R., Rubio K., Iglesias M., Vara I., Expósito A. (2018). Risk of Pelvic Inflammatory Disease After Intrauterine Insemination: A Systematic Review. *Reproductive Biomedicine Online*.

[4] Vichinsartvichai P. (2018). Bilateral Tubo-Ovarian Abscesses Presenting With Huge Pelvic Mass After Repeated Intrauterine Inseminations in a Woman With Severe Endometriosis. *The Journal of Obstetrics and Gynaecology Research*.

[5] Wadhwa L., Wadhwa S. N., Jindal S. (2015). A Rare Case of Flare-Up of PID in Infertility Treatment. *Case Reports in Obstetrics and Gynecology*.

[6] Ross J., Guaschino S., Cusini M., Jensen J. (2018). 2017 European Guideline for the Management of Pelvic Inflammatory Disease. *International Journal of STD & AIDS*.

[7] Workowski K. A., Bachmann L. H., Chan P. A. (2021). Sexually Transmitted Infections Treatment Guidelines, 2021. *MMWR Recommendations and Reports*.

[8] Dubova O., Zikan M. (2022). *Praktické repetitorium gynekologie a porodnictví*.

[9] Ness R. B., Soper D. E., Holley R. L. (2002). Effectiveness of Inpatient and Outpatient Treatment Strategies for Women With Pelvic Inflammatory Disease: Results From the Pelvic Inflammatory Disease Evaluation and Clinical Health (PEACH) Randomized Trial. *American Journal of Obstetrics and Gynecology*.

[10] Terao M., Koga K., Fujimoto A. (2014). Factors that Predict Poor Clinical Course Among Patients Hospitalized With Pelvic Inflammatory Disease. *The Journal of Obstetrics and Gynaecology Research*.

[11] Smeenk J., Wyns C., De Geyter C. (2023). O-153 Assisted Reproductive Technology (ART) in Europe 2020 and Development of a Strategy of Vigilance: Preliminary Results Generated From European Registers by the ESHRE EIM Consortium. *Human Reproduction*.

[12] Kreisel K., Torrone E., Bernstein K., Hong J., Gorwitz R. (2017). Prevalence of Pelvic Inflammatory Disease in Sexually Experienced Women of Reproductive Age - United States, 2013-2014. *MMWR. Morbidity and Mortality Weekly Report*.

[13] Sweet R. L. (1975). Anaerobic Infections of the Female Genital Tract. *American Journal of Obstetrics and Gynecology*.

[14] Mitchell C. M., Anyalechi G. E., Cohen C. R., Haggerty C. L., Manhart L. E., Hillier S. L. (2021). Etiology and Diagnosis of Pelvic Inflammatory Disease: Looking Beyond Gonorrhea and Chlamydia. *The Journal of Infectious Diseases*.

[15] Cohlen B., Bijkerk A., Van der Poel S., Ombelet W. (2018). IUI: Review and Systematic Assessment of the Evidence that Supports Global Recommendations. *Human Reproduction Update*.

[16] Kunz G., Beil D., Deininger H., Wildt L., Leyendecker G. (1996). The Dynamics of Rapid Sperm Transport Through the Female Genital Tract: Evidence From Vaginal Sonography of Uterine Peristalsis and Hysterosalpingoscintigraphy. *Human Reproduction*.

